# COVID-19 Pathology on Various Organs and Regenerative Medicine and Stem Cell-Based Interventions

**DOI:** 10.3389/fcell.2021.675310

**Published:** 2021-06-14

**Authors:** Babak Arjmand, Sepideh Alavi-Moghadam, Peyvand Parhizkar Roudsari, Mostafa Rezaei-Tavirani, Fakher Rahim, Kambiz Gilany, Fereshteh Mohamadi-Jahani, Hossein Adibi, Bagher Larijani

**Affiliations:** ^1^Cell Therapy and Regenerative Medicine Research Center, Endocrinology and Metabolism Molecular-Cellular Sciences Institute, Tehran University of Medical Sciences, Tehran, Iran; ^2^Metabolomics and Genomics Research Center, Endocrinology and Metabolism Molecular-Cellular Sciences Institute, Tehran University of Medical Sciences, Tehran, Iran; ^3^Proteomics Research Center, Shahid Beheshti University of Medical Sciences, Tehran, Iran; ^4^Health Research Institute, Thalassemia and Hemoglobinopathies Research Center, Ahvaz Jundishapur University of Medical Sciences, Ahvaz, Iran; ^5^Reproductive Immunology Research Center, Avicenna Research Institute, The Academic Center for Education, Culture and Research (ACECR), Tehran, Iran; ^6^Department of Biomedical Sciences, University of Antwerp, Antwerp, Belgium; ^7^Department of Integrative Oncology, Breast Cancer Research Center, Motamed Cancer Institute, ACECR, Tehran, Iran; ^8^Brain and Spinal Cord Injury Research Center, Neuroscience Institute, Tehran University of Medical Sciences, Tehran, Iran; ^9^Diabetes Research Center, Endocrinology and Metabolism Clinical Sciences Institute, Tehran University of Medical Sciences, Tehran, Iran; ^10^Endocrinology and Metabolism Research Center, Endocrinology and Metabolism Clinical Sciences Institute, Tehran University of Medical Sciences, Tehran, Iran

**Keywords:** coronavirus disease 2019, cytokine storm, mesenchymal stem cells, multi-organ failure, severe acute respiratory syndrome-coronavirus 2

## Abstract

Severe acute respiratory syndrome-coronavirus 2, a novel betacoronavirus, has caused the global outbreak of a contagious infection named coronavirus disease-2019. Severely ill subjects have shown higher levels of pro-inflammatory cytokines. Cytokine storm is the term that can be used for a systemic inflammation leading to the production of inflammatory cytokines and activation of immune cells. In coronavirus disease-2019 infection, a cytokine storm contributes to the mortality rate of the disease and can lead to multiple-organ dysfunction syndrome through auto-destructive responses of systemic inflammation. Direct effects of the severe acute respiratory syndrome associated with infection as well as hyperinflammatory reactions are in association with disease complications. Besides acute respiratory distress syndrome, functional impairments of the cardiovascular system, central nervous system, kidneys, liver, and several others can be mentioned as the possible consequences. In addition to the current therapeutic approaches for coronavirus disease-2019, which are mostly supportive, stem cell-based therapies have shown the capacity for controlling the inflammation and attenuating the cytokine storm. Therefore, after a brief review of novel coronavirus characteristics, this review aims to explain the effects of coronavirus disease-2019 cytokine storm on different organs of the human body. The roles of stem cell-based therapies on attenuating cytokine release syndrome are also stated.

## Introduction

In certain pathological conditions, such as a viral infection, the immune system may overproduce inflammatory cytokines. This situation sometimes can result in organ failure and death, known as a “cytokine storm.” Today, the cytokine storm has drawn further consideration because of the new pandemic disease related to the novel coronavirus [severe acute respiratory syndrome- coronavirus 2 (SARS-CoV-2)] or coronavirus disease 2019 (COVID-19) (Tisoncik et al., 2012; Cena and Chieppa, 2020; Fajgenbaum and June, 2020; Puelles et al., 2020; Song et al., 2020). Herein, COVID-19 has warned us of the essential role of high host immunity and the detrimental outcomes of immune dysregulation. Indeed, the cytokine storm in COVID-19 is known to be one of the key causes of multiple-organ dysfunction syndrome (MODS) or multi-organ failure (MOF) as a hallmark of COVID-19 severity (Wang and Ma, 2008; Zaim et al., 2020; Kim et al., 2021). Many elderly individuals and those with comorbidities are more prone to developing a cytokine storm and dysfunctional immune reactions (Ciabattini et al., 2020; Nidadavolu and Walston, 2020; Tay et al., 2020). In severe cases, the involvement of assorted organs finally ends up in protracting the hospitalization time and raising the share of mortality (Rieg et al., 2020). Acute lung failure, acute kidney damage, acute liver failure, cardiovascular disease, and a broad range of hematological anomalies along with neurological disorders are characterized by MOF and MODS. Accordingly, since the MOF and MODS in COVID-19 subjects are important health issues, developing the application of modern therapeutic solutions can lead to ameliorate results and reduce mortality rates (Mokhtari et al., 2020; Sherren et al., 2020). Nowadays, cell therapy and regenerative medicine as one of the assuring and modern therapies have been able to promote the function of organs involved in various disorders and trigger their real healing reactions. Hereupon, different cells, specifically various stem cells, can be applied (Wong et al., 2013; Monsel et al., 2014; Mao and Mooney, 2015; Goodarzi et al., 2019). Therefore, the purpose of the current review is to highlight the effects of cytokine storms on different organs in COVID-19 individuals and how stem cells manage this phenomenon.

## Characteristics of Novel Coronavirus and Coronavirus Disease 2019

Since December 2019, SARS-CoV-2 infection has led to a highly contagious disease named COVID-19 (Hu et al., 2020; Sun P. et al., 2020; Yang et al., 2020). Coronaviruses, as a member of the Coronavirinae subfamily (in the Coronaviridae family), are positive-sense, single-stranded, non-segmented, and enveloped RNA viruses (Arjmand et al., 2020; Harrison et al., 2020; Zamanian Azodi et al., 2020). This novel betacoronavirus has been shown 79% of genome sequence identity to the previous betacoronaviruses, including severe acute respiratory syndrome-CoV (SARS-CoV) and Middle East respiratory syndrome-CoV (MERS-CoV), which also caused fatal respiratory illnesses (Hu et al., 2020). The genome sequence of SARS-CoV-2 contains 14 open reading frames (ORFs) that 16 non-structural proteins (nsp) are encoded by two-thirds of that. Moreover, nine accessory proteins (ORF) plus four structural proteins are encoded by the remaining one-third (Harrison et al., 2020). Transmembrane spike (s) glycoprotein mediates the coronavirus entry, which is mentioned to be the main antibody target (Walls et al., 2020). Indeed, each S protein of SARS-CoV-2 has two subunits, S1 and S2 domains. Receptor-binding domain (within the S1 domain) is used by the virus to bind to the angiotensin-converting enzyme 2 (ACE2) as the cellular receptor. It could also promote the effects of transmembrane protease serine type 2 (TMPRSS2) on cleaving S protein (Dong et al., 2020). After binding, viral–host cell membrane fusion is activated leading to the release of viral RNA into the cytoplasm (Ni et al., 2020). ACE2 and TMPRSS2 can be expressed by different organs and tissues in addition to the lungs including the heart, kidney, colon, esophagus, liver, brain, testis, and gallbladder, which suggests the extrapulmonary effects of SARS-CoV-2 (Dong et al., 2020). On the other hand, COVID-19, according to its severity, can be classified into four types, namely, mild, moderate, severe, and critical, with different manifestations in each group. Herein, fever, fatigue, dry cough, and diarrhea are mentioned as the most common symptoms (Wang Y. et al., 2020). Upper respiratory tract-related symptoms can be seen in the mild form of the disease. Moderate patients also have cough, shortness of breath, and tachypnea symptoms with no severe form of symptoms. Acute respiratory distress syndrome (ARDS), septic shock, sepsis, severe dyspnea, and tachypnea are the signs and symptoms of severe pneumonia seen in the severe form of the disease. Moreover, in some of the patients, a critical disease can be developed with the manifestations of respiratory failure, septic shock, and MODS or MOF (Hassan et al., 2020). Droplet and human-to-human transmission as the direct ways and contaminated objects/airborne contagion as indirect means are the ways that SARS-CoV-2 can spread (Lotfi et al., 2020). Herein, the incubation period of COVID-19 ranges from 0 to 24 days, which is averagely estimated at around 6.4 days (Wang Y. et al., 2020). The most common radiological characteristics of COVID-19 are ground-glass opacities in the lungs, patchy consolidation, interlobular involvement, and alveolar exudates (Sahin et al., 2020). Moreover, laboratory studies have shown increased lactate dehydrogenase, C-reactive protein (CRP), erythrocyte sedimentation rate (ESR), total bilirubin levels, alanine aminotransferase (ALT), and aspartate aminotransferase (AST) as well as higher creatine kinase (CK) and D-dimer level. In addition, lymphocyte and eosinophil counts have shown lower levels as well as the levels of serum albumin and hemoglobin (Cascella et al., 2020; Velavan and Meyer, 2020). Additionally, as it was mentioned, MODS and systemic inflammatory response syndrome (SIRS) can be the results of SARS-CoV-2 infection in which a powerful cytokine storm has an important role (Sun X. et al., 2020).

## Cytokine Storm

It has been found that different triggers, including infections and malignancy, can result in unregulated host immune responses leading to the activated pathways of cytokine production. Thus, cytokine storm can be introduced as a systemic inflammation leading to the production of inflammatory cytokines and activation of immune cells (Tang Y. et al., 2020). Different immunity dysregulation disorders recognized by systemic inflammation, constitutional symptoms, and MOF (as the possible result of MODS) can be mentioned in this definition, too. Cytokine storm in different conditions can be varying in onset and duration, which depends on the cause and administered therapeutic approaches. However, the clinical manifestations of it usually overlap (Grupp et al., 2013; Fajgenbaum and June, 2020). Additionally, higher infiltration of cytokines has been seen in lung tissues of patients, too. In this regard, COVID-19 patients with severe conditions have shown more elevated pro-inflammatory cytokines compared to those in moderate conditions. Thus, it can be also related to the poor prognosis of the disease. Taken together, this can indicate the contribution of cytokine storm to the mortality of COVID-19 (Tang Y. et al., 2020). Therefore, it is essential to monitor patients for cytokine storm signs (Henderson et al., 2020).

### Clinical Features and Laboratory Abnormalities

Generally, cytokine storm can cause fever in almost all of the patients, which can be in high grades in severe patients. Cytokine storm can promote tissue damage pathways that, along with acute-phase physiological changes and immune cell-mediated reactions, may lead to some other symptoms, too. Some of these symptoms are fatigue, anorexia, rash, arthralgia, myalgia, headache, diarrhea, and neuropsychiatric changes (confusion, delirium, aphasia, and seizure). It can also lead to disseminated intravascular coagulation, hemostatic imbalance, catastrophic hemorrhages, hypotension, vasodilatory shock, and even death. It has been found that spontaneous hemorrhage is related to hyperinflammation, low platelet levels, and coagulopathy. Dyspnea, tachypnea, cough, pulmonary edema, ARDS, and hypoxemia are also possible respiratory symptom-related events that can be accompanied by cytokine storm. Splenomegaly and hepatomegaly, in addition to cardiac damage, liver injury, and renal failure, can be developed due to the more severe conditions of cytokine storm (Lee et al., 2014; Templin et al., 2015; Fajgenbaum and June, 2020; Gao et al., 2021). On the other hand, some laboratory findings of patients with cytokine storm syndrome are pancytopenia, abnormalities in liver function tests, increased triglyceride and ferritin, and lower fibrinogen levels. The cerebrospinal fluid analysis may also show some abnormalities in patients who have neurological manifestations (Rosado and Gopal, 2019). Exhaustion of natural killer cells/T cells leading to lymphopenia are other characteristics of chronic COVID-19 condition. Studies have shown that lymphocyte count is decreased significantly in severe COVID-19, and thus, lymphopenia can predict the disease prognosis and its clinical outcomes (Tan et al., 2020; Zhao et al., 2020). Cytokines impose a positive effect on the immune system to recruit immune cells to the inflammation sites. It could develop inflammation and some organ damages. Some of the important cytokines that have roles in this extreme activation are growth factors (GF), chemokines, interleukins (IL), tumor necrosis factor (TNF), interferons (IFN), and colony-stimulating factors (CSF). These cytokines, according to their functions, can be placed in two groups of pro-inflammatory and anti-inflammatory factors. For instance, TNF, IFN-γ, IL-1β, and IL-12 are in the pro-inflammatory group of factors, whereas transforming growth factor beta (TGF-β), IL-4, and IL-13 are some anti-inflammatory factors (Song et al., 2020). The concept of cytokine storm for COVID-19 infection is derived from the data that showed that critically ill patients have higher levels of TNFα, IFN gamma-induced protein-10 (IP-10), and the chemokine (C-C motif) ligand-2 (CCL2), compared to the patients in mild or moderate stages of the disease (Castelli et al., 2020). In addition to IP-10 and TNF-α, severely ill patients have shown higher levels of IL-2, IL-10, IL-7, granulocyte colony-stimulating factor (G-CSF), macrophage inflammatory protein-1A (MIP-1A), and monocyte chemoattractant protein-1 (MCP-1), too (Fara et al., 2020). In this regard, lower CD4, CD8, and natural killer (NK) T cell levels and increased monocyte and macrophage levels in COVID-19 patients can explain the higher levels of chemicals in them (Dong et al., 2020). IL-6 is also an important inflammatory cytokine that is elevated during COVID-19 inflammatory condition. It is useful for disease monitoring and can indicate the severity of the disease in its initial phases (Aziz et al., 2020; Liu et al., 2020). Chimeric antigen receptor T cell (CAR-T) treatment technology is introduced as a proper approach for immunotherapy (in infections and disorders such as hematological cancers). It can lead to cytokine release syndrome (CRS) as an adverse effect that is mainly caused by IL-6 (Bonifant et al., 2016; Kishimoto, 2021). Taken together, a valid immunological feature of COVID-19 is CRS in which hyper inflammation can manifest by a disrupted immune activation. Herein, an important association has been found between mortality rates and severe inflammation (Fara et al., 2020).

### Pathophysiological Features

In response to invading pathogens, the effective immune system is expected to return the body’s homeostasis by recognizing invaders and responding to them proportionally. In order to obtain this homeostasis, sufficient cytokine production is required along with the avoidance of hyperinflammatory reactions. Because, although inflammation activates innate and adaptive immune systems, it can cause collateral damages in hyperinflammatory states (Fajgenbaum and June, 2020). Endothelial dysfunction, metabolic abnormalities, and MOD can occur as the results of higher cytokine levels. Herein, elevated levels of TNF and IL-1β as acute phase response cytokines and higher IL-8 and MCP-1 levels can cause a facilitated increase of IL-6 levels (which, in COVID-19 subjects, is considered as a main intermediary of viral cytokine storm and inflammation). IL-6 in combination with membrane-bound IL-6 receptor or soluble IL-6 receptor forms a complex that affects glycoprotein 130 (gp130). Subsequently, it can lead to the regulated IL-6, granulocyte-macrophage colony-stimulating factor (GM-CSF), and MCP-1 and thus the perpetuation of the inflammatory responses. This process could be done through the Janus kinases (JAK)/signal-transducer and activator of transcription (STAT) pathway. The acute phase responses driven by IL-6 and other cytokines result in elevated ferritin, CRP, complement, and also pro-coagulant factors. On the other hand, the hyperinflammatory reactions of cytokine storm can produce reactive oxygen species (ROS) that causes cell death and stimulation of NLR family pyrin domain containing3 (NLRP3) and nuclear factor-kappa B (NF-κB) (Bhaskar et al., 2020). Herein, it has been found that transcription factor NF-κB can drive induced cytokine storm (Khalil et al., 2020). NLRP3 inflammasome can induce the immune responses leading to the increased release of cytokines and circulating cell debris, named danger-associated molecular pattern molecules (DAMPs). This can trigger and amplify the innate immunity reactions and the complement cascade (ComC) whose activation can be related to the worsened prognosis of COVID-19 patients (Ratajczak and Kucia, 2020). Pulmonary-activated platelets through the formation of platelet–neutrophil complexes (PNCs) have an important contribution to systemic sepsis, too. Herein, they are mentioned to be considerable sources of cytokines and ROS. Moreover, the effects of PNCs on increasing neutrophil recruitment and development of proinflammatory/procoagulant environment and their contribution to ARDS have been also found (Morris et al., 2020).

### Cytokine Storm and Multi-Organ Involvement in Coronavirus Disease 2019

**FIGURE 1:**
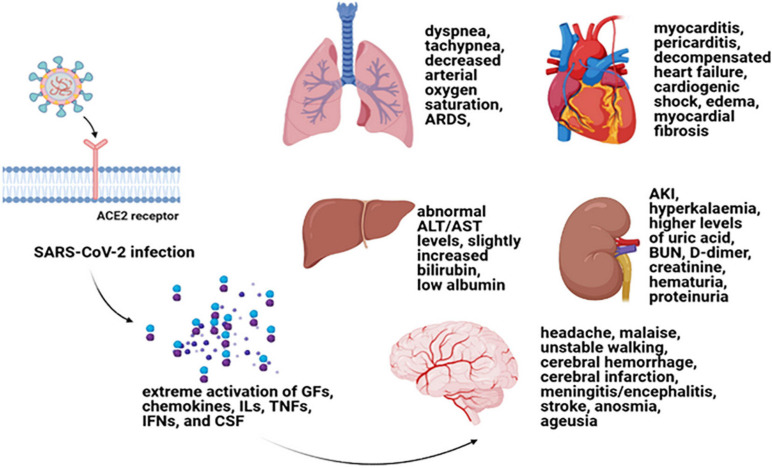
Cytokine storm and multiple-organ damages. Cytokine storm is the term used for a systemic inflammation that can lead to the extreme activation of GFs, chemokines, ILs, TNFs, IFN, and CSF (Song et al., 2020). Cytokine storm can result in MODS in COVID-19 patients with a worse prognosis. Myocarditis, pericarditis, decompensated heart failure, cardiogenic shock, edema, myocardial fibrosis as some cardiovascular complications (Ellison-Hughes et al., 2020; Vitiello and Ferrara, 2020); respiratory distress symptoms including dyspnea and tachypnea and decreased arterial oxygen saturation (Parhizkar Roudsari et al., 2020); renal damage symptoms such as AKI, hyperkalemia, higher levels of uric acid, BUN, D-dimer, and creatinine as well as hematuria and proteinuria (Kunutsor and Laukkanen, 2020; Patel et al., 2020; Puelles et al., 2020); an abnormal ALT or AST levels along with slightly increased bilirubin and low albumin (for liver damage consequences) (Sun J. et al., 2020; Wu J. et al., 2020); and headache, malaise, unstable walking as well as cerebral hemorrhage and cerebral infarction, meningitis/encephalitis, stroke, anosmia, and ageusia as some nervous system damages (Alomari et al., 2020; Berger, 2020) can be mentioned as some of the important organ involvements of COVID-19 due to cytokine storm. GF, growth factor; IL, interleukin; TNF, tumor necrosis factor; IFN, interferon; CSF, colony-stimulating factor; MODS, multiple-organ dysfunction syndrome; TGF-β, transforming growth factor beta; AKI, acute kidney injury; BUN, blood urea nitrogen; ALT, alanine aminotransferase; AST, aspartate aminotransferase.

Inappropriate reactions of host cells to a different group of acute insults can result in MODS, which is mentioned to be an important reason for mortality and morbidity in intensive care units (Wang and Ma, 2008). Indeed, in MODS condition, an autodestructive response of generalized inflammation can be the result of elevated pro-inflammatory cytokine levels (Aikawa, 1996). Hereupon, in COVID-19 infection, the hyperinflammatory responses along with severe acute respiratory syndrome direct effects are in association with disease complications (Zaim et al., 2020). The disease progression has been shown to be highly affected by extrapulmonary manifestations and its comorbidities. In this regard, multi-organ effects of COVID-19 have accompanied the infection since its emergence. Therefore, plausible organ injuries and comorbidities of COVID-19 require full attention in order to reduce death numbers (Sun X. et al., 2020; Wu T. et al., 2020; Zaim et al., 2020). Some important organ involvements of COVID-19 are explained separately in the next subtitles (Figure 1).

#### Cardiac Damage

Cardiovascular disease (CVD), as an important comorbidity accompanied by SARS-CoV-2 infection, can lead to a higher mortality rate than other associated comorbidities. Cytokine storm along with other mechanisms including thrombosis, endotheliosis, and lymphocytopenia can cause this cardiac damage or make it worsen (Unudurthi et al., 2020). Thus, the cardiovascular system can be involved in virus extrapulmonary effects with diverse manifestations of myocarditis, pericarditis, decompensated heart failure, cardiogenic shock, edema, myocardial fibrosis, and some other complications (Ellison-Hughes et al., 2020; Vitiello and Ferrara, 2020). The virus can cause its effects on the cardiac system through different ways of direct infection, binding to the functional receptors of ACE2, and immune damage. Cardiomyocyte infection by COVID-19 following by virus replication can lead to tissue degeneration, necrosis (Benítez-Guerra et al., 2020), and apoptosis (Heffernan et al., 2020). Indeed, the viral infection of COVID-19 can cause extracellular matrix remodeling leading to the production of fibrotic scars. These fibrotic lesions have some pathological effects that can even lead to death in patients including cardiac dysfunction and reduction of ejection fraction. Cardiac electrical conduction system can also experience some alterations that cause cardiac arrhythmias (Vitiello and Ferrara, 2020). The cardiac damage measured by increased levels of high-sensitivity troponin I has been mentioned to be found in about 20% of COVID-19 patients (Heffernan et al., 2020). Besides cardiac troponin I, increased levels of other laboratory cardiac markers have been seen in patients, such as higher levels of CK, creatine kinase-muscle/brain activity, myoglobin, alpha-hydroxybutyrate dehydrogenase (α-HBDH), N-terminal pro-brain natriuretic peptide (NT-proBNP), and AST (Benítez-Guerra et al., 2020). Cardiac markers are stated to be instrumental in cardiac damage diagnosis. In this regard, high-sensitivity troponin I is an appropriate marker for both diagnostic and prognostic approaches (Mishra et al., 2020). Early diagnosis of cardiac involvement is an essential step to utilize effective therapeutic approaches (Vitiello and Ferrara, 2020) due to the higher morbidity and mortality rates of patients with cardiac injury (Mishra et al., 2020).

#### Acute Respiratory Distress Syndrome

Acute respiratory distress syndrome is defined by an acute respiratory failure as a clinical syndrome in which arterial hypoxemia and dyspnea along with higher work of breathing can be presented. ARDS patients mostly need positive pressure ventilation and intubation. ARDS could be manifested in different disorders such as sepsis, pneumonia, major trauma, and aspiration (Matthay and Zemans, 2011). It has a broad range of causes and a wide spectrum of severity, imaging abnormalities, and other manifestations (Hariri and Hardin, 2020), which can overlap with some other conditions. Nonetheless, respiratory distress symptoms (for instance, dyspnea, and tachypnea); decreased arterial oxygen saturation; as well as epigastric pain, nausea/vomiting, hypotension, and fever are some mentionable symptoms (Parhizkar Roudsari et al., 2020). Hereupon, the associated symptoms of pneumonia-related COVID-19 range from asymptomatic (or mild upper respiratory tract infection) to severe forms of pneumonia, ARDS, and death (Xiao et al., 2020). Thus, ARDS is also an important and plausible comorbidity of COVID-19, the mentioned main cause of which is damages to the alveolar epithelial cells (Khalil et al., 2020). Cytokine storm, impaired IFN-I and IFN-III responses, and vasculopathy of COVID-19, as well as the host immune responses of COVID-19, can explain the underlying pathways of pneumonia-induced ARDS. The pro-inflammatory cascades of SARS-CoV-2 infection due to cytokine storm has a mentionable link to macrophage activation syndrome (MAS), which is a life-threatening feature of autoimmune diseases and can be mimicked in several viral infections. It can cause the damaged cytolytic activity of NK cells and CD8 + T cells. High levels of IL-6 may reflect an over-exuberant inflammatory reaction as the result of cytokine storm and can drive these impairments associated with MAS (Torres Acosta and Singer, 2020). Because of the rapid development of ARDS, the high mortality rate, and the lower quality of life among survivors, finding novel and more effective therapeutic approaches is considerably required (Xiao et al., 2020).

#### Renal Damage

Renal damage also takes a part in the extrapulmonary effects of COVID-19 that has been observed in a significant population of COVID-19-infected patients. Autopsy studies have also shown renal involvement due to COVID-19 infection. In this regard, acute kidney injury (AKI) is a common finding that can be observed in up to 25% of critically ill patients of COVID-19 (with underlying comorbidities) (Patel et al., 2020). Needing for renal replacement therapy and electrolyte disturbance, such as hyperkalemia, are other common renal complications of COVID-19 (Kunutsor and Laukkanen, 2020). Renal damage can be manifested by higher levels of uric acid, blood urea nitrogen (BUN), D-dimer, and creatinine as well as hematuria and proteinuria (Puelles et al., 2020). Indeed, proteinuria can be the presentation of patients at hospital admission, and AKI mostly develops in critically ill patients at later stages of COVID-19. Thus, it could be a marker for MOD and the severity of the disease (Ronco et al., 2020). Taken together, it has been mentioned that renal damage is related to the severe forms of COVID-19 infection with fatal outcomes, and pre-existing CKD can lead to a higher incidence of AKI. A high expression of ACE2, TMPRSS2, and cathepsin L (CTSL) in the kidneys and direct cytopathic effects of the virus are possible causes of kidney involvement during the COVID-19 pandemic (Naicker et al., 2020; Puelles et al., 2020). Hypovolemia and ARDS-related AKI are other possible mechanisms of kidney injury associated with COVID-19. Moreover, cytokine storm in association with secondary hemophagocytic lymphohistiocytosis (sHLH) has a significant role in renal involvement of COVID-19. Hemodynamic changes, hypercoagulable state, and direct effects of cytokines (such as IL-6 and TNF) can be caused by hyperinflammatory states of cytokine storm that may lead to acute tubular necrosis (ATN) and tubulointerstitial nephritis (TIN) (Ahmed et al., 2020). There are no specific therapeutic options for AKI-related COVID-19, and intensive care along with clinical experience are mainly supportive that declares the requirement for developing new approaches for the management of this involvement (Ronco et al., 2020).

#### Liver Damage

There are numerous studies that have shown liver involvement due to COVID-19 infection. Although the exact underlying mechanism of liver damage has not been founded yet, abnormal ALT or AST levels along with slightly increased bilirubin are common manifestations of liver dysfunction (Wu J. et al., 2020). Higher liver enzymes are found more commonly in males. In addition, severe cases have more elevated liver enzymes than milder cases of the disease. Low albumin is also an indicator of severe infection with a worse prognosis. According to the effects of cytokine storm on liver damage, the inflammation biomarkers such as CRP, serum ferritin, lactate dehydrogenase (LDH), IL-6, IL-2, and D-dimer were significantly higher in severely ill patients of COVID-19 (Sun J. et al., 2020). Results of an autopsy pathological study have shown moderate microvascular steatosis that was along with mild inflammation of the lobular portal zone, but direct killing influences of the virus were not observed (Wu J. et al., 2020). Taken together, direct viral effects on bile ducts, the role of cholangiocytes (for instance with their cell entry receptor of ACE2), and immune system activation particularly cytokine storm could be the probable causes for liver injury (Alqahtani and Schattenberg, 2020). On the other hand, drug-induced liver injury and hypoxia-induced damage are other possible mechanisms that can be hypothesized for liver damage because of SARS-CoV-2 infection (Kudaravalli et al., 2020; Wu J. et al., 2020). It should be mentioned that patients with a past medical history of liver diseases are more susceptible to liver damage from SARS-CoV-2 (Sun J. et al., 2020). However, more in-depth studies are required to demonstrate the causes of liver damage and to provide anti-COVID-19 treatments that particularly work on liver function (Vitiello et al., 2021).

#### Central Nervous System Damage

Besides the typical symptoms of COVID-19, some patients may show some neurological symptoms such as headache and malaise. Unstable walking as well as cerebral hemorrhage and cerebral infarction, meningitis/encephalitis, stroke, anosmia, and ageusia are other possible effects of SARS-CoV-2 infection on the nervous system (Alomari et al., 2020; Berger, 2020). Conducted studies have shown that neurological symptoms may be manifested in more than one-third of patients with COVID-19. However, they may be more commonly seen in severe infections (Niazkar et al., 2020). It has been shown that the virus can infect neurons and reduce synapse formation between them in which the olfactory route and blood–brain barrier could be the possible routes of invasion (Iadecola et al., 2020a; Marshall, 2020). *In vivo* studies done on human ACE2 transgenic mice found that an ACE2-dependent manner of neuronal infection can lead to neuronal death due to the organoids. Viral proteins and molecular complexes of damaged cells could enter the compromised blood–brain barrier, and after brain entry, they act as DAMPs and pathogen-associated molecular patterns (PAMPs). This can promote innate immune responses and express Toll-like receptors (TLR). It was found that these receptors can mediate the SARS-CoV pro-inflammatory effects that may result in higher cytokine production and impaired brain function (Iadecola et al., 2020a). Hypothalamic–pituitary–adrenocortical (HPA) axis can be also activated due to the unregulated cytokines in COVID-19, which can cause the autonomic nervous system and catecholamine/steroids release (Iadecola et al., 2020b). Despite all this, pathophysiological mechanisms underlying CNS-related COVID-19 infection should be found more precisely (Divani et al., 2020).

## Treatments for Cytokine Storms

Overall, important strategies to avoid the development of cytokine storms and ameliorate the prognosis of infection include the reduction of viral load by targeted therapeutic approaches in the early stages of the disease (with no or moderate symptoms) and the regulation of inflammatory reactions via immune modulators (Florindo et al., 2020; Khadke et al., 2020; Pan et al., 2020; Tang L. et al., 2020; Ye et al., 2020). Herein, cytokine inhibition, blood purification medical care, corticosteroid therapies, and cell-based approaches are the foremost therapeutic strategies (Iannaccone et al., 2020; Ye et al., 2020). Accordingly, cytokine inhibition, e.g., by means of IL-6/IL-6R blockers, IL-1 family blockers, TNF-α blockers, and IFN-αβ blockers; blood purification medical care through the adsorption, plasma exchange, perfusion, and filtration of blood/plasma; corticosteroid therapies, which contribute to histone acetyltransferase (HAT) inhibition and histone deacetylase 2 (HDAC2) interest recruitment to be able to downregulate inflammatory genes; and cell-based approaches have powerful anti-inflammatory and immune-regulatory roles (Choudhary et al., 2020; Henriksen, 2020; Hojyo et al., 2020; Iannaccone et al., 2020).

## Regenerative Medicine and Cell-Based Treatments

**FIGURE 2:**
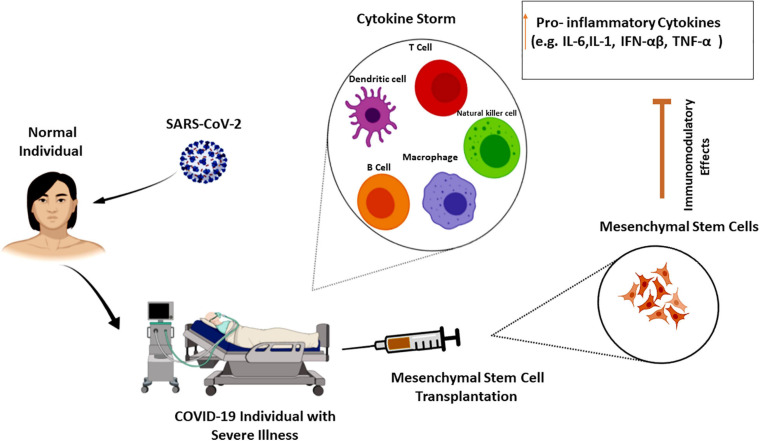
Mesenchymal stem cells effects on cytokine storm. Cytokine storm as the hallmark of COVID-19 severity is the increasing level of pro-inflammatory cytokines that can lead to organ failure and death. The immune-regulatory effects of mesenchymal stem cells on various cell types of the innate and adaptive immune system can attenuate cytokine storm in severe COVID-19 cases (Lee and Song, 2018; Wang et al., 2018; Zhu et al., 2020).

Cell therapy and regenerative medicine are marked as one of the most hopeful possible strategies for the regeneration of damaged or failed tissue and organs in the medical system. There are different approaches here, containing the use of cells from both autologous and allogeneic sources (Saberi et al., 2008; Aghayan et al., 2014a; Goodarzi et al., 2014, 2015). In other words, it encompasses a wide range of treatments via using various types of cells (e.g., T cells, NK lymphocytes, and different stem cells) with varying outcomes. In this context, the adoptive T-cell therapy or CAR T-cell therapy approach as a kind of immunotherapy has been shown to be effective against some infections and diseases. Herein, T cells from patient’s own immune system (autologous source) are extracted and sent to a lab for genetic modification. The patient is then re-infused with the engineered cells (Maus et al., 2014; Bonifant et al., 2016; Maus and Levine, 2016; Seif et al., 2019). Despite the impressive effectiveness of CAR T-cell therapy in the treatment, it has a number of serious side effects including CRS and neurologic difficulties. CRS with an immediate onset tends to be a cytokine storm (Chen et al., 2019; Hong et al., 2021). Currently, T-cell therapy has also shown promise in immunosuppressed individuals as a preventive measure against COVID-19. Accordingly, investigators employed peripheral blood cells from convalescent subjects who had been endangered by the virus (Keller et al., 2020). Regulatory T cell-related strategies have been also suggested as considered treatment approaches for disease management according to their capacity for inactivation of innate/adaptive immunity through inhibitory molecules (Stephen-Victor et al., 2020). Additionally, transferring modified/unmodified antigen-specific T cells has shown promising results in the treatment of different disorders by reconstituting T cell subsets (effector/memory cells). In this context, adoptive T cell therapy by transferring T cell immune subsets is mentioned to have therapeutic benefits that can be the same as adult tissue stem cell features. However, high maintenance of memory T cells required and engraftment processes may create some limitations (Busch et al., 2016). In this regard, specific COVID-19-related T cells (within CD45RA- memory T cells) have been recognized that can be feasibly received by CD45RA depletion from convalescent donors. These cells can provide a population of cells for lymphopenia condition along with quick reactions to infection. COVID-19 CD45RA- memory T cells also provide immunity against secondary probable infections that may be found in COVID-19-hospitalized individuals (Ferreras et al., 2021). HLA-matched cytotoxic T cells isolated from convalescent patients are other promising approaches for the treatment of COVID-19 same to EBV-specific cytotoxic T cells, which were utilized for EBV + -related lymphomas (Hanley et al., 2020). Another promising candidate for significant advancement has been NK cell therapy. Hereupon, autologous or allogeneic origins may be used to create pure populations of NK cells. Using the allogeneic NK cells as a platform for CAR engineering has risen due to the limitations of autologous NK cells (such as decreased effector role and the demand for a patient-specific stock) (Veluchamy et al., 2017; Daher and Rezvani, 2018). Since a decrease in the number of NK cells can be linked to the severity of the COVID-19 infection, some clinical trials used engineered NK cells to help battle COVID-19 (Market et al., 2020; van Eeden et al., 2020). However, using NK cell also has a number of drawbacks that may hinder their effectiveness. Short lifespan (in the lack of cytokine support), low cell numbers, and vulnerability to the immunosuppressive situation, all of which could limit their trafficking and operation (Nayyar et al., 2019; Liu et al., 2021). In accordance with various introduced limitations, as the epicenter of regenerative medicine, mesenchymal stem cells (MSCs) have been widely investigated and applied, and also have appeared throughout this area as a strong and commonly used cell source. Their capacity to differentiate into diverse cell lineages, migration, and cellular regulator secretion together with immunosuppressive and immunomodulatory potential of MSC secretome are the features that make them extremely valuable. On the other hand, their isolation is almost easy and does not have significant ethical concerns (Aghayan et al., 2014b; Larijani et al., 2014, 2015, 2021; Goodarzi et al., 2018a,b; Payab et al., 2018; Arjmand et al., 2019; Abedi et al., 2020; Tayanloo-Beik et al., 2021). These features makes them the most suitable stem cell approaches among many of them (Azmi et al., 2020). The umbilical cord, adipose tissue bone marrow, dental pulp, and menstrual blood are important sources of MSCs. MSCs derived from adipose tissue have been mentioned to have more interesting results initially, but the best source of stem cell is required to be found yet (Song et al., 2021). Moreover, through their impacts on T and B cells, macrophages, and dendritic cells, they help regenerate and refresh the condition (Lee and Song, 2018; Wang et al., 2018). Accordingly, by inhibiting the proliferation of T and B cells and by successful regulation of pro-inflammatory cytokines to optimize the microenvironment for intrinsic recovery, MSCs can reduce the cytokine storm. On the other hand, as the indirect effects to attenuate cytokine storm, they can restrict the innate immune system cell infiltration and consequently decrease the secretion of inflammatory cytokines (Ellison-Hughes et al., 2020; Gupta et al., 2020; Zhu et al., 2020; Jeyaraman et al., 2021; Figure 2). 6 days after MSC therapy, cytokine storm-related immunity cells were showed to have dwindled. Increased levels of lymphocytes and regulatory dendritic cells along with decreased CRP; IL-1, 6, and 12; IFN-γ; and TNF levels are also other results of this kind of therapy. Indeed, MSCs can provide antimicrobial peptides and anti-inflammatory cytokines (Leng et al., 2020; Rajarshi et al., 2020; Wang H.C. et al., 2020). Besides these anti-inflammatory features, secretion of IL-10 and some growth factors along with their regeneration and reparative capacity make them a potent therapeutic approach for lung repair and ARDS treatment in early stages (Azmi et al., 2020). MSC administration has been also shown to have benefits in sepsis and septic shock conditions regarding their capacity to normalize inflammatory biomarkers, oxygen saturation, and pulmonary improvements on CT imaging. For sepsis condition, umbilical cord-derived MSCs [especially from Wharton’s jelly (WJ)], due to their effectiveness and acceptability, are mentioned to be the best source for MSCs (Laroye et al., 2020). In this respect, the US Food and Drug Administration (FDA) has recently confirmed the safety and efficacy of MSCs for widespread application in COVID-19 cases (Choudhery and Harris, 2020; Kavianpour et al., 2020). In addition to the mentioned benefits of MSC administration, there are still some challenges; MSC-related features regarding their dosage, route of administration, frequency, and homing into the damaged sites have provided some limitations. Remaining ethical concerns along with lack of standardized protocols in preparation and isolation processes are other challenges. One other important concern about MSC therapy is the side effect of increased hypercoagulability (Jeyaraman et al., 2021). Thus, according to the higher risk of thrombosis, cell-free therapies including MSC secretome and MSC extracellular vesicles (EVs) seem to be interesting treatment approaches for COVID-19 that have shown no risk of mutagen/oncogenicity. Exosomes harbor different types of miRNAs/mRNAs and diverse protein components and have lower accompanied risk and decreased infection transmission. However, dosage, timing, and route of cell delivery are required to be known more clearly. Their capability for nebulized delivery and their longer storage periods also make them promising alternative therapeutic approaches (Maron-Gutierrez and Rocco, 2020; Kheirkhah et al., 2021). Therein, a number of cell-based clinical trials for COVID-19 are reviewed in Table 1.

**TABLE 1:** Number of cell-based clinical trials for COVID-19 (https://clinicaltrials.gov/).

**Clinical trial heading**	**Applied cell**	**Thetotal number of participants**	**State of the recruitment**	**ClinicalTrials.gov identifier**	**Locations**
Mesenchymal Stem Cell Infusion for COVID-19 Infection	Mesenchymal stem cells	20	Recruiting	NCT04444271	Pakistan
Mesenchymal Stem Cell for Acute Respiratory Distress Syndrome Due for COVID-19 (COVID-19)	Mesenchymal Stem cells	10	Recruiting	NCT04416139	Mexico
Safety and Efficacy of Mesenchymal Stem Cells in the Management of Severe COVID-19 Pneumonia	Umbilical cord-derived mesenchymal stem cells	30	Not yet recruiting	NCT04429763	United States
Novel Coronavirus Induced Severe Pneumonia Treated by Dental Pulp Mesenchymal Stem Cells	Dental pulp mesenchymal stem cells	24	Not yet recruiting	NCT04302519	China
Mesenchymal Stem Cells in Patients Diagnosed With COVID-19	Mesenchymal stem cells	20	Recruiting	NCT04611256	United States
Use of Mesenchymal Stem Cells in Acute Respiratory Distress Syndrome Caused by COVID-19	Mesenchymal stem cells derived from Wharton’s jelly of umbilical cords	9	Active, not recruiting	NCT04456361	United States
Efficacy of Infusions of MSC From Wharton Jelly in the SARS-Cov-2 (COVID-19) Related Acute Respiratory Distress Syndrome	*Ex vivo* expanded Wharton’s jelly mesenchymal stem cells	30	Not yet recruiting	NCT04625738	France
Mesenchymal Stem Cell Therapy for SARS-CoV-2-related Acute Respiratory Distress Syndrome	Mesenchymal stem cells	60	Recruiting	NCT04366063	Iran
Novel Adoptive Cellular Therapy With SARS-CoV-2 Specific T Cells in Patients With Severe COVID-19	Adoptive T-cell therapy	8	Recruiting	NCT04351659	Singapore
Mesenchymal Stem Cells Therapy in Patients With COVID-19 Pneumonia	Mesenchymal stem cells	21	Completed	NCT04713878	Turkey
Part Two of Novel Adoptive Cellular Therapy With SARS-CoV-2 Specific T Cells in Patients With Severe COVID-19	SARS-CoV-2-specific T cells	18	Recruiting	NCT04457726	Singapore
A Study of Cell Therapy in COVID-19 Subjects With Acute Kidney Injury Who Are Receiving Renal Replacement Therapy	Allogeneic human mesenchymal stromal cells	22	Recruiting	NCT04445220	United States
Safety of T Regulatory Cell Therapy in Subjects With COVID-19 Induced Acute Respiratory Distress Syndrome	T regulatory cells	20	Not yet recruiting	NCT04737161	United States
Cell Therapy Using Umbilical Cord-derived Mesenchymal Stromal Cells in SARS-CoV-2-related ARDS	Umbilical cord Wharton’s jelly derived human mesenchymal stromal cells	47	Active, not recruiting	NCT04333368	France
Treatment of Coronavirus COVID-19 Pneumonia (Pathogen SARS-CoV-2) With Cryopreserved Allogeneic P_MMSCs and UC-MMSCs	Cryopreserved placenta-derived mesenchymal stromal cells	30	Recruiting	NCT04461925	Ukraine
Study of Intravenous Administration of Allogeneic Adipose Stem Cells for COVID-19	Adipose-derived allogeneic mesenchymal stem cell	20	Recruiting	NCT04486001	United States
A Randomized, Double-Blind, Placebo-Controlled Clinical Trial to Determine the Safety and Efficacy of Hope Biosciences Allogeneic Mesenchymal Stem Cell Therapy (HB-adMSCs) to Provide Protection Against COVID-19	Allogeneic adipose-derived mesenchymal stem cells	100	Active, not recruiting	NCT04348435	United States
Mesenchymal Stromal Cell Therapy for Severe Covid-19 Infection	Bone marrow-derived mesenchymal stromal Cells	20	Recruiting	NCT04445454	Belgium
Treatment of COVID-19 Patients Using Wharton’s jelly Mesenchymal Stem Cells	Umbilical cord Wharton’s jelly derived human mesenchymal stem cells	5	Recruiting	NCT04313322	Jordan
A Phase I/II Study of Universal Off-the-shelf NKG2D-ACE2 CAR-NK Cells for Therapy of COVID-19	NK cells	90	Recruiting	NCT04324996	China
Safety and Efficacy of Allogeneic Human Dental Pulp Mesenchymal Stem Cells to Treat Severe COVID-19 Patients	Allogeneic human dental pulp mesenchymal stem cells	20	Recruiting	NCT04336254	China
Treatment With Human Umbilical Cord-derived Mesenchymal Stem Cells for Severe Corona Virus Disease 2019 (COVID-19)	Umbilical cord-derived mesenchymal stem cells	100	Completed	NCT04288102	China
Clinical Research of Human Mesenchymal Stem Cells in the Treatment of COVID-19 Pneumonia	Umbilical cord mesenchymal stem cells	30	Recruiting	NCT04339660	China
Cell Therapy Using Umbilical Cord-derived Mesenchymal Stromal Cells in SARS-CoV-2-related ARDS	Umbilical cord Wharton’s jelly derived human mesenchymal stromal cells	47	Active, not recruiting	NCT04333368	France
Study of Human Umbilical Cord Mesenchymal Stem Cells in the Treatment of Novel Coronavirus Severe Pneumonia	Umbilical cord mesenchymal stem cells	48	Not recruiting	NCT04273646	China
Mesenchymal Stem Cell Treatment for Pneumonia Patients Infected With 2019 Novel Coronavirus	Mesenchymal stem cells	20	Recruiting	NCT04252118	China
Umbilical Cord (UC)-Derived Mesenchymal Stem Cells (MSCs) Treatment for the 2019-novel Coronavirus (n COV) Pneumonia	Umbilical cord mesenchymal stem cells	16	Recruiting	NCT04269525	China
NK Cells Treatment for Novel Coronavirus Pneumonia	NK cells	30	Recruiting	NCT04280224	China

## Conclusion and Future Scope

Due to the prevalence and complications of COVID-19, including cytokine storm, which is followed by organ dysfunction or failure and death, finding the efficient approach to treat and improve patients is of great importance. Cell therapy is now a modern way of treating several diseases, and many experiments have been performed in recent months to use different types of cells to treat the COVID-19, i.e., the MSC transplantation (Parhizkar Roudsari et al., 2020). Manifold aspects associated with the MSC application (e.g., standard protocols for isolation and harvesting, selection of the proper source for isolation, the appropriate dosage, route, and the ideal timing of delivery) should be more discussed. Herein, in explaining the potential of MSCs, preclinical researches and continuing randomized trials will perform an important part to promote our knowledge about MSCs’ fight against SARS-CoV-2 (Mastrolia et al., 2019; Al-Khawaga and Abdelalim, 2020). Because of possible accompanying side effects and limitations of stem cell-based therapeutic approaches, EVs are emerging alternative cellular treatments that have some advantages over MSCs (Maron-Gutierrez and Rocco, 2020). On the other hand, the importance of conducting more extensive studies to better understand the new coronavirus and its different variants cannot be ignored. This opens the door to researchers for designing more effective treatments. Accordingly, genetically modified MSCs have the ability to solve the challenges as a new sector and a developing field (Sage et al., 2016).

## Author Contributions

SA-M and PP wrote the first draft of the manuscript. FM-J, MR-T, and HA helped to study and gather information. FR and KG extensively edited the manuscript. BL participated in a critical review. BA helped to supervise the project and gave final approval of the version to be published. All authors contributed to the study’s conception and design.

## Conflict of Interest

The authors declare that the research was conducted in the absence of any commercial or financial relationships that could be construed as a potential conflict of interest.

## GLOSSARY

SARS-CoV-2, severe acute respiratory syndrome coronavirus-2; COVID-19, coronavirus disease-2019; SARS-CoV, severe acute respiratory syndrome coronavirus; MERS-CoV, Middle East respiratory syndrome coronavirus; ORFs, open reading frames; nsp, non-structural proteins; S, spike; ACE2, angiotensin converting enzyme 2; TMPRSS2, transmembrane protease serine type 2; ARDS, acute respiratory distress syndrome; MODS, multiple-organ dysfunction syndrome; MOF, multi-organ failure; CRP, C-reactive protein; ESR, erythrocyte sedimentation rate; ALT, alanine aminotransferase; AST, aspartate aminotransferase; CK, creatine kinase; SIRS, systemic inflammatory response syndrome; GF, growth factor; IL, interleukin; TNF, tumor necrosis factor; IFN, interferon; CSF, colony-stimulating factor; TGF-β, transforming growth factor beta; IP-10, IFN gamma-induced protein-10; CCL2, chemokine (C-C motif) ligand-2; MIP-1A, macrophage inflammatory protein-1A; MCP-1, monocyte chemoattractant protein-1; CRS, cytokine release syndrome; G-CSF, granulocyte colony-stimulating factor; GM-CSF, granulocyte-macrophage colony-stimulating factor; JAK: Janus kinases; STAT, signal-transducer and activator of transcription; ROS, reactive oxygen species; NF-κB, nuclear factor-kappa B; DAMPs, danger-associated molecular pattern molecules; ComC, complement cascade; PNCs, platelet–neutrophil complexes; CVD, cardiovascular disease; α-HBDH, alpha-hydroxybutyrate dehydrogenase; NT-proBNP, N-terminal pro-brain natriuretic peptide; MAS, macrophage activation syndrome; AKI, acute kidney injury; BUN, blood urea nitrogen; CTSL, cathepsin L; sHLH, hemophagocytic lymphohistiocytosis; ATN, acute tubular necrosis; TIN, tubulointerstitial nephritis; PAMPs, pathogen-associated molecular patterns; TLR, Toll-like receptors; HPA, hypothalamic–pituitary–adrenocortical; HAT, histone acetyltransferase; HDAC2, histone deacetylase 2; FDA, Food and Drug Administration; MSCs, mesenchymal stem cells; WJ, Wharton’s jelly; EVs, extracellular vesicles; NK, natural killer; CAR, chimeric antigen receptor.
